# Nutritional correlates of monetary diet cost in young, middle-aged and older Japanese women

**DOI:** 10.1017/jns.2017.18

**Published:** 2017-05-22

**Authors:** Keiko Shiraki, Kentaro Murakami, Hitomi Okubo, M. Barbara E. Livingstone, Satomi Kobayashi, Hitomi Suga, Satoshi Sasaki, The Three-Generation Study of Women on Diets and Health Study Group

**Affiliations:** 1 Department of Nutrition, School of Human Cultures, University of Shiga Prefecture, Shiga, Japan; 2 Department of Social and Preventive Epidemiology, School of Public Health, University of Tokyo, Tokyo, Japan; 3 Department of Health Promotion, National Institute of Public Health, Saitama, Japan; 4 Northern Ireland Centre for Food and Health, Ulster University, Coleraine, UK; 5 Department of Social and Preventive Epidemiology, Graduate School of Medicine, University of Tokyo, Tokyo, Japan

**Keywords:** Diet, Monetary cost, Women, Japan, BDHQ, brief diet history questionnaire, DHQ, diet history questionnaire, EI, energy intake, PAL, physical activity level

## Abstract

Studies in many Western countries have consistently shown that monetary diet cost is positively associated with diet quality, but this may not necessarily be the case in Japan. This cross-sectional study examined the nutritional correlates of monetary diet cost among 3963 young (all 18 years old), 3800 middle-aged (mean age 48 years) and 2211 older (mean age 74 years) Japanese women. Dietary intakes were assessed using a comprehensive self-administered diet history questionnaire for young and middle-aged women and a brief self-administered diet history questionnaire for older women. Monetary diet cost was estimated using retail food prices. Total vegetables, fish and shellfish, green and black tea, white rice, meat, fruit and alcoholic beverages contributed most (79–89 %) to inter-individual variation in monetary diet cost. Multiple regression analyses showed that monetary diet cost was negatively associated with carbohydrate intake, but positively with intakes of all other nutrients examined (including not only dietary fibre and key vitamins and minerals but also saturated fat and Na) in all generations. For food group intakes, irrespective of age, monetary diet cost was associated inversely with white rice and bread but positively with pulses, potatoes, fruit, total vegetables, fruit and vegetable juice, green and black tea, fish and shellfish, and meat. In conclusion, in all three generations of Japanese women and contrary to Western populations, monetary diet cost was positively associated with not only healthy dietary components (including fruits, vegetables, fish and shellfish, dietary fibre, and key vitamins and minerals), but also less healthy components (including saturated fat and Na).

Although food choice is influenced by many factors including taste, convenience, concerns about nutrition and body weight, the price of food is clearly an important determinant^(^^1^^,^^2^^)^. In general, foods that are energy-dense but nutrient-poor such as fat and oils, and sugar and confectioneries, provide dietary energy at lowest cost, whereas the price of foods that are nutrient-dense such as fish and shellfish, vegetables and fruits is much higher^(^^3^^–^^7^^)^.

Previous studies from the UK^(^^4^^,^^8^^,^^9^^)^, Spain^(^^3^^,^^10^^)^, France^(^^6^^,^^11^^,^^12^^)^, the Netherlands^(^^13^^)^ and the USA^(^^5^^,^^14^^–^^18^^)^ all suggest that healthier diets cost more. Typically, higher-quality diets are characterised by higher intakes of whole grains, lean meats, fruits and vegetables, and by lower intakes of fats and sugars, and refined grains^(^^19^^,^^20^^)^. Further, the positive association between monetary diet cost and diet quality may explain the socio-economic gradient in health widely observed in Western countries^(^^7^^,^^8^^,^^20^^–^^22^^)^, with higher socio-economic status associated with better health^(^^23^^,^^24^^)^.

However, in Japan, where unclear or inverse associations between socio-economic status and health status are observed^(^^25^^,^^26^^)^, the association between monetary diet cost and diet quality may be more complex than that observed in Western countries. Indeed, in selected Japanese population groups, monetary diet cost was associated not only with healthier (including higher intakes of dietary fibre, key vitamins and minerals, fruits, vegetables and lower dietary energy density) but also less healthy diets (including higher intakes of saturated fat and Na)^(^^27^^–^^29^^)^.

Further investigation in a general Japanese population is clearly needed because the relationship between monetary diet cost and diet quality is an important issue. Therefore, the aim of the present cross-sectional study was to examine nutritional correlates of monetary diet cost with food and nutrient intake in young, middle-aged and older Japanese women. Specifically, we investigated the food contributions to diet cost, the inter-individual variation in diet cost explained by each food group, and the association between diet cost and nutrient and food group intake after adjustment for a range of potential confounding factors.

## Subjects and methods

### Survey design

The present cross-sectional study was based on data from the Three-Generation Study of Women on Diets and Health, a questionnaire survey conducted in northern and western Japan in 2011 and in eastern Japan in 2012. The study design and survey procedure have been described in detail^(^^30^^)^. Briefly, two questionnaires on dietary habits and lifestyle factors were distributed to a total of 7016 dietetic students from eighty-five higher education institutions in thirty-five of forty-seven prefectures in Japan during the orientation session or the first lecture designed for freshmen in April 2011 or 2012. Each student was also requested to invite his/her mother and grandmother to join the study and to distribute similar questionnaires to those agreeing to take part. Recruitment priority for the grandmother generation was given to the maternal or, if unavailable, paternal grandmother, followed by his/her female acquaintance aged 65–89 years. In total, 4933 students, including 4656 women and 277 men (response rate 70·3 %), 4044 mothers (57·6 %), and 2332 women from the grandmothers’ generation (33·2 %) answered both questionnaires.

This study was conducted according to the guidelines laid down in the Declaration of Helsinki and all procedures involving human subjects were approved by the Ethics Committee of the University of Tokyo Faculty of Medicine. Written informed consent was obtained from each subject and also from a parent/guardian for subjects aged <20 years.

### Analytic sample

In the present study, we considered that the students (excluding males), mothers and grandmothers (including acquaintances) consisted of groups of young, middle-aged and older women, respectively.

For the youngest group, we selected female students aged 18 years (*n* 4065). We then excluded those not only living in eastern Japan but who also participated in the 2011 survey (because it was assumed that they could not report their usual dietary habits and lifestyle due to the occurrence of the Great East Japan Earthquake in March 2011) (*n* 39), those who answered the questionnaires after 19 May to minimise the influence of dietetic education (*n* 56) and those whose data were derived from the institution where the response rate was extremely low (2 %) (*n* 2). We further excluded those with missing information on the variables of interest (*n* 5).

For the analysis of middle-aged women, we selected mothers aged 34 to 60 years (*n* 4012). We then excluded those not only living in eastern Japan but who also participated in the 2011 survey (*n* 63) and those whose data were derived from the institution with the low response rate (*n* 2). We further excluded those with erroneous or missing information on the variables of interest (*n* 147).

For the analysis of older women, we selected grandmothers (and acquaintances) aged 61 to 94 years (*n* 2325). We then excluded those not only living in eastern Japan but who also participated in the 2011 survey (*n* 47) and those whose data were derived from the institution with the low response rate (*n* 1). We further excluded those with erroneous or missing information on the variables of interest (*n* 66). Consequently, the final sample sizes were 3963, 3800 and 2211 for young, middle-aged and older women, respectively.

### Dietary assessment

Dietary habits during the preceding month were assessed using a comprehensive diet history questionnaire (DHQ)^(^^31^^–^^33^^)^ for young and middle-aged women and a brief diet history questionnaire (BDHQ)^(^^31^^,^^32^^)^ for older women. Responses to the DHQ and BDHQ, as well as to the lifestyle questionnaire, were checked at least once by survey staff at the study centre. If any missing or erroneous responses were given to questions which were considered essential for analysis, the participant was asked to complete these again. Details of the structure and calculation method of dietary intake of the DHQ and BDHQ have been published elsewhere^(^^31^^–^^33^^)^. Briefly, the DHQ and BDHQ are structured, self-administered questionnaires that ask about the consumption frequency (and portion size in DHQ) of selected foods commonly consumed in Japan, as well as general dietary behaviour and usual cooking methods^(^^33^^,^^34^^)^. Estimates of the daily intake for foods (151 items in DHQ and fifty-eight items in BDHQ), energy and selected nutrients were calculated using an *ad hoc* computer algorithm for the DHQ and BDHQ, which was based on the Standard Tables of Food Composition in Japan^(^^35^^)^. To minimise the influence of dietary misreporting^(^^36^^,^^37^^)^, energy-adjusted values of dietary variables were calculated based on the density method (i.e. percentage of energy for energy-providing nutrients and amount per 4184 kJ of energy for other nutrients and foods). A relative validity of the DHQ and BDHQ has been previously investigated among ninety-two women aged 31–69 years using a 16-d weighed dietary record as reference^(^^31^^,^^32^^)^. Briefly, for the DHQ, the median value of Spearman's correlation coefficients for food groups was 0·43 (range −0·09 to 0·77), and that of Pearson's correlation coefficients for nutrients was 0·57 (range 0·27 to 0·87). The corresponding values for the BDHQ were 0·44 (range 0·14 to 0·82) and 0·54 (range 0·34 to 0·87), respectively.

### Calculation of monetary diet cost

Monetary diet cost (Japanese yen/d) was calculated by multiplying the amount of each food item consumed (derived from the DHQ (g/d) for young and middle-aged women or the BDHQ (g/d) for older women) by estimated price of food (Japanese yen/g) and summing the products. The procedure for estimating costs was based on the assumption that all foods were purchased and then prepared and consumed at home, with some exceptions (French fries and doughnuts) described below. Sale prices were not used to determine costs. Costs of composite foods such as pizza were calculated using prices of frozen equivalents. Calculation included correction for preparation and waste.

The price of food items was obtained from two sources. The first was the National Retail Price Survey of 2004^(^^38^^)^. This survey was conducted in 167 villages, towns and cities, and average prices were calculated as mean value of all survey areas, weighted for population size. The second source was information on price from the websites of supermarkets (Seiyu, Japan) and fast-food restaurants (McDonalds, Japan and Mister Donut, Japan) located throughout Japan. For French fries and doughnuts, the price was determined by that from fast-food restaurants, as these items are usually purchased in such restaurants in Japan.

To determine the price of individual food items, we used the following three procedures:

1. Direct matching. Each food in the DHQ and BDHQ was directly matched to foods appearing in the National Retail Price Survey. This procedure was used to determine the price of 179 of the 236 items in the DHQ (76 %) and 121 of the 150 in the BDHQ (81 %).
2. Comparable food items. When exact matching to a food appearing in the National Retail Price Survey was not possible, the closest alternative was chosen. This procedure was used to determine the price of forty-two items in the DHQ (18 %) and twenty-seven items in the BDHQ (18 %).
3. Website. When no comparable food in the National Retail Price Survey was available, prices were taken from the websites. This procedure was used to determine the price of fifteen items in the DHQ (6 %) and two in the BDHQ (1 %).

The monetary cost (Japanese yen/100 g) of each food item (as well as the percentage contribution to total cost in the present populations) is listed in Supplementary Table S1 for DHQ and Supplementary Table S2 for BDHQ. As crude monetary diet cost was highly correlated with energy intake (EI) in the present populations (Pearson correlation coefficient 0·81 to 0·90), energy-adjusted monetary diet cost by the density method (Japanese yen/4184 kJ) was used in the present study. A detailed description of the cost calculation methods has been published elsewhere^(^^27^^,^^39^^)^. In a group of ninety-two Japanese women aged 31–69 years, Pearson's correlation coefficient between DHQ and 16-d weighed dietary records was 0·60^(^^39^^)^, and the correlation for BDHQ was 0·44 (S Sasaki and K Murakami, unpublished results), suggesting satisfactory reliability of the DHQ and BDHQ in terms of monetary diet cost.

The food price database was created using data collected in 2004, whereas the present data were collected in 2011 and 2012. Despite this, a correction for inflation was not considered necessary since it is the relative, not absolute, costs within the population that are of interest for the purposes of the present study^(^^8^^)^.

### Assessment of other variables

All the variables used were based on each participant's self-reported information. Age at the time of the survey was calculated based on birth date. Residential area was grouped into six regions (Hokkaido and Tohoku, Kanto, Hokuriku and Tokai, Kinki, Chugoku and Shikoku, or Kyushu) and also into three categories according to population size (city with a population ≥1 million, city with a population <1 million, or town and village). Living status (not considered in middle-aged women because almost all lived with their family) was grouped into three categories (living alone, living with family, or living with others), but for older women those living with others were added to those living with family because of the very small number of subjects (*n* 4). BMI was calculated as body weight (kg) divided by square of body height (m). Weight status was grouped into three categories: underweight (BMI <18·5 kg/m^2^), normal weight (BMI ≥18·5 to <25 kg/m^2^) and overweight (BMI ≥25·0 kg/m^2^)^(^^40^^)^. All the results did not change when using the Asian BMI cut-offs recommended by the WHO^(^^41^^)^ (data not shown). Information on current smoking (yes or no), current alcohol drinking (yes or no), dietary supplement use (not only vitamins/minerals but also specialty supplements such as botanicals, other herbs, etc.; yes or no) and prescription medicine use (yes or no) was also used. Eating out was categorised as ≤3 times/month, once per week, 2–3 times/week, or ≥4 times/week (not available in older women). Physical activity was computed as the average total metabolic equivalent-hours score per d on the basis of the frequency and duration of seven activities (walking, bicycling, standing, running, high-intensity activities, sleeping, and sedentary activity) over the preceding months^(^^42^^)^, which was categorised into quartiles. For only middle-aged women, occupation was considered (housewife, part-time job, or full-time job). Except for young women, education level was categorised as low, middle, and high (≤12 years, 13–15 years, and ≥16 years for middle-aged women and ≤9 years, 10–12 years, and ≥13 years for older women, respectively). Current marital status (yes or no) was also considered for middle-aged and older women.

Misreporting of EI was evaluated on the basis of the ratio of EI:BMR (the Goldberg cut-off)^(^^43^^)^. Subjects were identified as plausible, under- and over-reporters of EI according to whether the individual's ratio was within, below or above the 95 % confidence limits for agreement between EI:BMR and the respective physical activity level (PAL). In the present analysis, the PAL for sedentary lifestyle (i.e. 1·55)^(^^43^^)^ was applied for all subjects. This decision was not only because of a lack of a validated measure of physical activity but also because in all generations, time spent on sedentary activity (range of mean: 11·65 to 14·00 h/d) was predominant compared with other activities: walking (1·31 to 1·95 h/d), bicycling (0·14 to 0·31 h/d), standing (1·64 to 2·99 h/d), running (0·02 to 0·04 h/d), high-intensity activities (0·05 to 0·06 h/d) and sleeping (6·22 to 7·97 h/d). BMR was estimated according to an equation specifically developed for Japanese women, as follows: BMR (kJ/d) = (0·0481 × body weight (kg) + 0·0234 × body height (cm)  − 0·0138 × age (years)  − 0·9708)^(^^44^^,^^45^^)^. The 95 % confidence limits for agreement (upper and lower cut-off values) between EI:BMR and the PAL were calculated, taking into account CV in intakes and other components of energy balance (i.e. the within-subject variation in EI: 23 %; the precision of the estimated BMR relative to the measured BMR: 8·5 %; and the between-subject variation in PAL: 15 %)^(^^43^^)^. Consequently, under-, plausible and over-reporters were defined as having EI:BMR <1·09, 1·09–2·21 and >2·21, respectively^(^^43^^)^.

### Statistical analysis

All statistical analyses were performed for young, middle-aged and older women separately, using SAS statistical software version 9.3 (SAS Institute Inc.). All reported *P* values are two-tailed, and *P* < 0·05 was considered statistically significant. Descriptive data are presented as means and standard deviations for continuous variables, except for food group intakes for which medians and 25th and 75th percentiles are used, and numbers and percentages of subjects for categorical variables. Differences in monetary diet cost across categories of selected characteristics were examined by ANOVA.

Using the PROC REG procedure, stepwise forward regression analyses were carried out to investigate the contribution of the selected twenty food groups for young and middle-aged women and eighteen food groups for older women to the inter-individual variation in monetary diet cost. The significance level for entry into the model was set at *P* < 0·1. For those food groups contributing at least 1 % variation, multiple regression analyses were performed (using the PROC REG procedure) with predictive food groups as explanatory variables and monetary diet cost as the response variable.

Multiple regression analyses were performed to explore the association of monetary diet cost (the explanatory variable) with food group and nutrient intakes (response variables). Using the PROC REG procedure, we calculated the adjusted regression coefficients (with standard errors) of variation of intakes of selected food groups and nutrients by a 100 Japanese yen increase in monetary diet cost (per 4184 kJ). Potential confounding factors considered included survey year, residential block, size of residential area, weight status, current smoking, current alcohol drinking, dietary supplement use, medication use, physical activity and dietary reporting status. Further adjustment was made for living status and eating out in young women; eating out, occupation, education, current marital status and age (<45, 45–49, or ≥50 years) in middle-aged women; and living status, education, current marital status and age (61–69, 70–74, 75–79, or ≥80 years) in older women. These analysis were repeated after excluding under- and over-reporters. Monetary diet cost was analysed continuously after confirming the linearity of relationships using quintile categories.

## Results

### Population characteristics

All of the young women were 18 years old, whereas mean age was 47·7 (sd 3·9) years (range 34–60 years) in middle-aged women and 74·4 (sd 5·2) years (range 61–94 years) in older women (Table 1). Mean EI:BMR was 1·47 (sd 0·50) in young women, 1·66 (0·49) in middle-aged women, and 1·86 (sd 0·61) in older women. Mean value of energy-adjusted monetary diet cost was 479 (sd 98) Japanese yen/4184 kJ for young women, 531 (sd 101) Japanese yen/4184 kJ for middle-aged women, and 629 (sd 105) Japanese yen/4184 kJ for older women.

**Table 1.** Basic and dietary characteristics of young, middle-aged and older Japanese women (Arithmetic mean values and standard deviations or median values and 25th and 75th percentiles)

	Young (*n* 3963)		Middle-aged (*n* 3800)		Older (*n* 2211)	
	Arithmetic mean	sd	Arithmetic mean	sd	Arithmetic mean	sd
Age (years)	18	0	47·7	3·9	74·4	5·2
Body height (cm)	157·7	5·3	157·3	5·1	150·4	5·5
Body weight (kg)	52·0	7·8	54·5	8·2	51·6	7·9
BMI (kg/m^2^)	20·9	2·8	22·0	3·1	22·8	3·2
EI (kJ/d)	7242	2399	7717	2231	7369	2277
EI:BMR	1·47	0·50	1·66	0·49	1·86	0·61
Monetary diet cost						
Crude diet cost (Japanese yen/d)*	838	351	981	339	1115	417
Energy-adjusted diet cost (Japanese yen/4184 kJ)*	479	98	531	101	629	105
Nutrient intake						
Protein (% of energy)	13·0	2·2	13·7	2·0	16·9	3·2
Total fat (% of energy)	29·4	6·3	29·1	5·9	25·7	5·1
Saturated fat (% of energy)	8·3	2·3	8·0	2·0	6·7	1·6
Monounsaturated fat (% of energy)	10·4	2·7	10·4	2·5	8·9	2·0
Polyunsaturated fat (% of energy)	6·5	1·6	6·7	1·5	6·4	1·4
Carbohydrate (% of energy)	56·3	7·3	54·2	7·1	55·9	7·4
Alcohol (% of energy)	0·1	0·7	1·8	4·1	0·5	2·3
Dietary fibre (g/4184 kJ)	6·2	2·0	6·7	2·0	8·0	2·2
Cholesterol (mg/4184 kJ)	171	75	165	57	231	76
Na (mg/4184 kJ)	2134	639	2279	618	2558	526
K (mg/4184 kJ)	1030	280	1194	276	1631	399
Ca (mg/4184 kJ)	246	94	267	93	367	112
Mg (mg/4184 kJ)	113	28	131	27	159	33
Fe (mg/4184 kJ)	3·6	0·9	3·7	0·9	5·1	1·2
Vitamin A (μg/4184 kJ)†	271	206	288	185	476	258
Vitamin E (mg/4184 kJ)‡	4·1	1·2	4·2	1·1	4·5	1·0
Folate (μg/4184 kJ)	147	52	156	48	239	75
Vitamin C (mg/4184 kJ)	46·5	23·5	47·9	23·2	85·5	31·2
Food intake (g/4184 kJ)§						
White rice	148·3	98·8, 201·5	132·1	87·9, 178·4	159·9	116·6, 210·4
Other grainsǁ¶	0	0, 0	0	0, 0	–	–
Noodles	28·3	13·1, 49·2	27·9	13·5, 46·4	25·0	13·9, 41·1
Bread	14·6	6·2, 25·8	15·4	7·0, 27·1	17·4	6·8, 32·9
Other grain products¶**	4·3	0, 10·4	3·5	0, 9·9	–	–
Pulses	19·6	11·3, 34·4	27·9	17·5, 43·1	42·9	28·5, 61·6
Potatoes	12·5	8·1, 19·7	12·9	8·5, 21·3	28·1	14·7, 45·3
Sugar and confectioneries	49·6	35·3, 67·8	44·6	31·6, 60·1	37·5	21·4, 56·1
Fat and oil	11·9	8·7, 16·1	11·7	8·8, 15·0	8·2	5·6, 10·9
Fruit	16·0	8·0, 31·0	21·8	10·1, 44·0	55·2	29·0, 87·4
Total vegetables	97·4	64·1, 139·9	114·2	81·8, 155·7	168·2	126·0, 225·1
Alcoholic beverages	0	0, 0	0	0, 33·1	0	0, 0
Fruit and vegetable juice	3·6	0, 32·8	0	0, 15·3	0	0, 18·8
Green and black tea	261·2	122·4, 444·9	233·0	108·2, 382·1	256·2	141·4, 349·9
Coffee	0	0, 13·1	135·8	59·0, 239·4	58·0	4·5, 110·1
Soft drinks	23·9	2·2, 65·0	7·5	0, 35·4	0·0	0, 8·1
Fish and shellfish	22·8	14·3, 32·7	28·0	19·9, 38·3	54·4	36·0, 75·0
Meat	33·3	22·8, 46·7	34·2	23·8, 46·7	30·4	20·0, 41·5
Eggs	18·2	8·3, 30·2	16·0	9·1, 26·5	17·7	11·4, 28·3
Dairy products	37·0	11·8, 85·0	54·1	18·3, 99·8	66·3	24·5, 98·5

EI, energy intake.

* 1 Japanese yen = 0·0099 US dollars = 0·0088 Euros = 0·0074 British pounds (September 2016).

† Retinol equivalents.

‡ α-Tocopherol.

§ Median values and 25th and 75th percentiles

ǁ Including white rice mixed with barley, white rice with germ, half-milled rice, 70 % milled rice and brown rice.

¶ Not available for older women because the brief-type diet history questionnaire used for this age group did not include these food groups.

** Including pizza, Japanese-style pancakes and cornflakes.

### Percentage contribution of each food group to monetary diet cost

The highest contributor to monetary diet cost differed by generations: meat (17·2 %) in young women, total vegetables (15·9 %) in middle-aged women, and fish and shellfish (22·4 %) in older women (Table 2). However, in all generations, the top four contributors were the same: meat, total vegetables, sugar and confectioneries, and fish and shellfish (54·8 to 60·3 % in total). Other important contributors included white rice (5·8 to 7·3 %) and green and black tea (5·0 to 7·7 %) in all generations, alcoholic beverages (4·8 %) in middle-aged women, and fruit (6·7 %) in older women.

**Table 2.** Percentage contribution of each food group to monetary diet cost in young, middle-aged and older Japanese women* (Ranks; arithmetic mean values and 95 % confidence intervals)

	Young (*n* 3963)			Middle-aged (*n* 3800)			Older (*n* 2211)		
	Rank	Arithmetic mean	95 % CI	Rank	Arithmetic mean	95 % CI	Rank	Arithmetic mean	95 % CI
Meat	1	17·2	16·9, 17·4	2	15·0	14·8, 15·2	3	10·7	10·5, 11·0
Total vegetables	2	14·8	14·6, 15·1	1	15·9	15·7, 16·1	2	16·6	16·3, 16·8
Sugar and confectioneries	3	14·2	13·9, 14·4	4	11·3	11·1, 11·5	4	10·6	10·3, 10·9
Fish and shellfish	4	11·7	11·5, 11·9	3	12·7	12·5, 12·8	1	22·4	22·0, 22·8
Green and black tea	5	7·7	7·5, 7·9	5	6·1	6·0, 6·3	7	5·0	4·8, 5·1
White rice	6	7·3	7·1, 7·4	6	5·8	5·7, 5·9	6	6·0	5·8, 6·1
Dairy products	7	4·2	4·0, 4·3	8	4·7	4·6, 4·8	9	2·3	2·2, 2·3
Fruit	8	3·5	3·4, 3·6	10	3·8	3·7, 3·9	5	6·7	6·5, 6·8
Pulses	9	2·8	2·7, 2·8	11	3·3	3·2, 3·4	8	3·1	3·0, 3·1
Noodles	10	2·5	2·4, 2·6	12	2·2	2·1, 2·2	13	1·6	1·5, 1·6
Soft drinks	11	2·5	2·3, 2·6	14	1·6	1·5, 1·7	18	0·5	0·4, 0·6
Potatoes	12	1·8	1·8, 1·9	15	1·5	1·5, 1·6	11	2·2	2·1, 2·3
Bread	13	1·8	1·7, 1·8	13	1·6	1·6, 1·7	10	2·2	2·1, 2·3
Fat and oil	14	1·7	1·6, 1·7	16	1·4	1·3, 1·4	17	0·6	0·6, 0·6
Eggs	15	1·7	1·6, 1·7	17	1·3	1·3, 1·3	14	1·2	1·2, 1·2
Fruit and vegetable juice	16	1·3	1·2, 1·4	19	0·7	0·6, 0·7	16	0·8	0·7, 0·9
Other grain products†‡	17	1·2	1·1, 1·2	18	1·0	0·9, 1·0	–	–	–
Other grains‡§	18	0·7	0·7, 0·8	20	0·6	0·6, 0·7	–	–	–
Coffee	19	0·5	0·4, 0·5	9	4·1	4·0, 4·2	12	1·6	1·5, 1·7
Alcoholic beverages	20	0·1	0·1, 0·2	7	4·8	4·5, 5·1	15	1·0	0·8, 1·2

* Food groups are listed in descending order of their arithmetic mean percentage contribution to monetary diet cost in young women.

† Including pizza, Japanese-style pancakes and cornflakes.

‡ Not available for older women because the brief-type diet history questionnaire used for this age group did not include these food groups.

§ Including white rice mixed with barley, white rice with germ, half-milled rice, 70 % milled rice and brown rice.

### Contribution of food groups to inter-individual variation in monetary diet cost

Total vegetables, fish and shellfish, green and black tea, white rice (only young and older women), meat, fruit and alcoholic beverages (only middle-aged and older women) contributed most (79 to 89 %) to inter-individual variation in monetary diet cost (Table 3). Other contributors included soft drinks (3 %), sugar and confectioneries (3 %), and fruit and vegetable juice (1 %) in young women, soft drinks (3 %), sugar and confectioneries (3 %), and coffee (4 %) in middle-aged women, and sugar and confectioneries (1 %) in older women.

**Table 3.** Food groups contributing to the inter-individual variation in monetary diet cost (Japanese yen/4184 kJ) in young, middle-aged and older Japanese women* (Regression coefficients with their standard errors and partial determination coefficients)

	Young (*n* 3963)			Middle-aged (*n* 3800)			Older (*n* 2211)		
	β†	se†	Partial *R*^2^	β†	se†	Partial *R*^2^	β†	se†	Partial *R*^2^
Model *R*^2^		0·88			0·89			0·90	
Total vegetables	6·62	0·09	0·38	7·03	0·10	0·27	5·30	0·10	0·22
Fish and shellfish	21·41	0·35	0·14	19·84	0·36	0·09	19·86	0·26	0·45
Green and black tea	1·23	0·02	0·12	1·14	0·02	0·06	1·27	0·04	0·04
White rice	−1·11	0·08	0·08	–‡	–‡	–‡	−1·91	0·15	0·10
Meat	16·29	0·30	0·05	17·14	0·32	0·06	11·87	0·44	0·02
Fruit	6·82	0·21	0·03	7·20	0·19	0·04	5·40	0·18	0·03
Soft drinks	1·88	0·06	0·03	2·62	0·09	0·03	–‡	–‡	–‡
Sugar and confectioneries	7·19	0·23	0·03	7·50	0·26	0·03	6·19	0·37	0·01
Fruit and vegetable juice	1·99	0·09	0·01	–‡	–‡	–‡	–‡	–‡	–‡
Alcoholic beverages	–‡	–‡	–‡	5·34	0·05	0·27	4·91	0·16	0·03
Coffee	–‡	–‡	–‡	1·23	0·03	0·04	–‡	–‡	–‡

β, Regression coefficient.

* Food groups listed are those contributing at least 1 % of the variation of monetary diet cost based on the stepwise regression analysis with twenty food groups (white rice; other grains; noodles; bread; other grain products; pulses; potatoes; sugar and confectioneries; fat and oil; fruit; total vegetables; fruit and vegetable juice; alcoholic beverages; green and black tea; coffee; soft drinks; fish and shellfish; meat; eggs; and dairy products) in young and middle-aged women and eighteen food groups (white rice; noodles; bread; pulses; potatoes; sugar and confectioneries; fat and oil; fruit; total vegetables; fruit and vegetable juice; alcoholic beverages; green and black tea; coffee; soft drinks; fish and shellfish; meat; eggs; and dairy products) in older women as explanatory variables and monetary diet cost as the response variable.

† Model with listed variables as explanatory variables and monetary diet cost as the response variable; regression coefficients can be interpreted as the change of monetary diet cost (Japanese yen/4184 kJ) with a 10 g increase in intake of each food group (per 4184 kJ). 1 Japanese yen = 0·0099 US dollars = 0·0088 Euros = 0·0074 British pounds (September 2016).

‡ Not contributing at least 1 % of the variation of monetary diet cost.

### Associations between monetary diet cost and selected characteristics

Monetary diet cost differed significantly according to many of the selected characteristics examined (Supplementary Table S3). For residential block, monetary diet cost was the highest in Kinki and the lowest in Hokkaido and Tohoku for young women, but the highest in Kanto and the lowest in Hokuriku and Tokai for older women. For living status in young women, monetary diet cost was the highest for those living with family and the lowest for those living alone. Size of residential area and education were positively associated with monetary diet cost (except for young women). Among current smokers a higher mean value of monetary diet cost was observed only in middle-aged women. Current alcohol drinkers had a lower mean value of monetary diet cost in middle-aged women but a higher mean value in older women. Supplement users had a higher mean value of monetary diet cost in young and older women but a lower value in middle-aged women. Medication users had a higher mean value of monetary diet cost in young women but a lower value in older women. Physical activity was positively associated with monetary diet cost in young and older women. For dietary reporting status, over-reporters had a higher monetary diet cost compared with under- and plausible reporters (except for middle-aged women). Age was positively associated with monetary diet cost in only middle-aged women (data not shown).

### Associations between monetary diet cost and nutrient intakes

Associations of monetary diet cost with nutrient intakes are shown in Table 4. After adjustment for potential confounding factors, monetary diet cost was negatively associated with total carbohydrate intake in all generations. Conversely, monetary diet cost was positively associated with intakes of all other nutrients examined, namely protein, total, saturated, monounsaturated and polyunsaturated fats, dietary fibre, Na, K, Ca, Mg, Fe, vitamins A, E and C, and folate as well as cholesterol and alcohol intakes.

**Table 4.** Associations of monetary diet cost (Japanese yen/4184 kJ) with nutrient intakes in young, middle-aged and older Japanese women* (Regression coefficients with their standard errors)

	Young (*n* 3963)			Middle-aged (*n* 3800)			Older (*n* 2211)		
	β†	se†	Standardised β	β†	se†	Standardised β	β†	se†	Standardised β
Protein (% of energy)	1·31	0·03	0·59	0·94	0·03	0·48	2·32	0·04	0·76
Total fat (% of energy)	2·04	0·10	0·32	0·82	0·10	0·14	1·80	0·10	0·37
Saturated fat (% of energy)	0·55	0·04	0·24	0·18	0·03	0·09	0·35	0·03	0·23
Monounsaturated fat (% of energy)	0·85	0·04	0·31	0·39	0·04	0·16	0·57	0·04	0·30
Polyunsaturated fat (% of energy)	0·58	0·03	0·34	0·32	0·02	0·21	0·47	0·03	0·35
Carbohydrate (% of energy)	−2·95	0·11	−0·39	−2·77	0·10	−0·39	−3·99	0·12	−0·57
Alcohol (% of energy)	0·04	0·01	0·06	1·32	0·06	0·32	0·23	0·04	0·10
Dietary fibre (g/4184 kJ)	1·14	0·03	0·56	0·87	0·03	0·43	1·12	0·04	0·55
Cholesterol (mg/4184 kJ)	16·02	1·26	0·21	9·66	0·93	0·17	35·82	1·37	0·50
Na (mg/4184 kJ)	235·6	10·3	0·36	198·2	9·9	0·32	293·6	8·7	0·59
K (mg/4184 kJ)	219·3	3·2	0·76	184·0	3·4	0·67	287·3	5·3	0·76
Ca (mg/4184 kJ)	37·2	1·48	0·39	27·2	1·5	0·30	67·1	1·8	0·63
Mg (mg/4184 kJ)	17·2	0·38	0·61	16·3	0·4	0·60	24·9	0·4	0·79
Fe (mg/4184 kJ)	0·64	0·01	0·68	0·43	0·01	0·51	0·83	0·02	0·75
Vitamin A (μg/4184 kJ)‡	84·5	3·2	0·40	57·9	3·0	0·31	96·4	5·0	0·39
Vitamin E (mg/4184 kJ)§	0·74	0·02	0·62	0·46	0·02	0·44	0·70	0·02	0·71
Folate (μg/4184 kJ)	40·8	0·6	0·76	31·7	0·6	0·66	46·1	1·2	0·65
Vitamin C (mg/4184 kJ)	16·3	0·3	0·68	12·4	0·3	0·54	18·4	0·5	0·62

β, Regression coefficient.

* Adjustment was made for survey year, residential block, size of residential area, weight status, current smoking, current alcohol drinking, dietary supplement use, medication use, physical activity and dietary reporting status. For young women, further adjustment was made for living status and eating out. For middle-aged women, further adjustment was made for eating out, occupation, education, current marital status and age. For elderly women, further adjustment was made for living status, education, current marital status and age.

† Regression coefficients can be interpreted as the change in nutrient intakes with 100 Japanese yen increase in monetary diet cost (per 4184 kJ). 1 Japanese yen = 0·0099 US dollars = 0·0088 Euros = 0·0074 British pounds (September 2016). All values are statistically significant (*P* = 0·0001 for alcohol in young women and P < 0·0001 for all others).

‡ Retinol equivalents.

§ α-Tocopherol.

### Associations between monetary diet cost and food group intakes

Table 5 presents associations between monetary diet cost and food group intakes with adjustment for potential confounding factors. Monetary diet cost was inversely associated with intakes of white rice, noodles (except for middle-aged women), bread, other grain products (not available for older women) and dairy products (only middle-aged women). There was an inverse association between monetary diet cost and sugar and confectionery intake in middle-aged and older women, with there being a positive association in young women. Monetary diet cost was also positively associated with intakes of all other food groups examined, including pulses, potatoes, fat and oil (except for middle-aged women), fruit, total vegetables, alcoholic beverages (only middle-aged women), fruit and vegetable juice, green and black tea, coffee (except for older women), soft drinks (except for older women), fish and shellfish, meat, and eggs (except for middle-aged women). The same associations between monetary diet cost and intakes of food groups (except for no associations with eggs in young women and with sugar and confectioneries, fat and oil, and eggs in older women) and nutrients were observed after excluding under- and over-reporters of EI (*n* 1001 in young women, *n* 715 in middle-aged women, and *n* 662 in older women) (data not shown).

**Table 5.** Associations of monetary diet cost (Japanese yen/4184 kJ) with intakes of food groups in young, middle-aged and older Japanese women* (Regression coefficients with their standard errors)

	Young (*n* 3963)				Middle-aged (*n* 3800)				Older (*n* 2211)			
	β†	se†	*P*	Standardised β	β†	se†	*P*	Standardised β	β†	se†	*P*	Standardised β
White rice	−32·9	1·2	<0·0001	−0·40	−25·2	1·1	<0·0001	−0·35	−35·7	1·1	<0·0001	−0·54
Other grains‡§	–ǁ	–ǁ	–ǁ	–ǁ	–ǁ	–ǁ	–ǁ	–ǁ	–	–	–	–
Noodles	−2·7	0·6	<0·0001	−0·08	−0·6	0·5	0·19	−0·02	−1·5	0·5	0·002	−0·07
Bread	−4·1	0·3	<0·0001	−0·24	−4·1	0·3	<0·0001	−0·25	−3·3	0·3	<0·0001	−0·21
Other grain products§¶	−0·7	0·1	<0·0001	−0·08	−0·6	0·1	<0·0001	−0·08	–	–	–	–
Pulses	7·4	0·5	<0·0001	0·24	4·5	0·5	<0·0001	0·16	6·8	0·5	<0·0001	0·27
Potatoes	3·2	0·2	<0·0001	0·26	2·7	0·2	<0·0001	0·22	4·2	0·5	<0·0001	0·18
Sugar and confectioneries	1·9	0·5	<0·0001	0·07	−1·8	0·4	<0·0001	−0·08	−1·7	0·5	0·001	−0·07
Fat and oil	0·8	0·1	<0·0001	0·11	0·1	0·1	0·23	0·02	0·2	0·1	0·02	0·05
Fruit	9·5	0·4	<0·0001	0·35	7·7	0·5	<0·0001	0·25	14·1	0·8	<0·0001	0·34
Total vegetables	43·9	0·9	<0·0001	0·65	34·3	0·9	<0·0001	0·55	43·9	1·4	<0·0001	0·57
Alcoholic beverages	–ǁ	–ǁ	–ǁ	–ǁ	45·3	1·5	<0·0001	0·41	–ǁ	–ǁ	–ǁ	–ǁ
Fruit and vegetable juice	8·6	1·0	<0·0001	0·14	4·1	0·6	<0·0001	0·11	3·9	0·9	<0·0001	0·10
Green and black tea	118·9	4·4	<0·0001	0·41	75·8	3·8	<0·0001	0·32	36·9	3·5	<0·0001	0·22
Coffee	10·7	1·2	<0·0001	0·15	29·7	2·6	<0·0001	0·18	0·0	1·8	0·99	−0·0003
Soft drinks	6·6	1·5	<0·0001	0·07	5·8	1·0	<0·0001	0·09	1·7	1·1	0·12	0·03
Fish and shellfish	8·6	0·2	<0·0001	0·52	7·0	0·2	<0·0001	0·44	20·4	0·5	<0·0001	0·69
Meat	7·1	0·3	<0·0001	0·36	4·9	0·3	<0·0001	0·27	4·2	0·4	<0·0001	0·24
Eggs	0·7	0·3	0·02	0·04	0·3	0·2	0·16	0·02	0·8	0·3	0·003	0·07
Dairy products	0·2	1·2	0·89	0·00	−1·9	1·1	<0·0001	−0·03	1·2	1·2	0·28	0·02

β, Regression coefficient.

* Adjustment was made for survey year, residential block, size of residential area, weight status, current smoking current alcohol drinking, dietary supplement use, medication use, physical activity and dietary reporting status. For young women, further adjustment was made for living status and eating out. For middle-aged women, further adjustment was made for eating out, occupation, education, current marital status and age. For elderly women, further adjustment was made for living status, education, current marital status and age.

† Regression coefficients can be interpreted as the change in food group intakes (g/4184 kJ) with 100 Japanese yen increase in monetary diet cost (per 4184 kJ). 1 Japanese yen = 0·0099 US dollars = 0·0088 Euros = 0·0074 British pounds (September 2016).

‡ Including pizza, Japanese-style pancakes and cornflakes.

§ Not available for older women because the brief-type diet history questionnaire used for this age group did not include these food groups.

ǁ Not examined because more than 75 % of the subjects were non-consumers.

¶ Including white rice mixed with barley, white rice with germ, half-milled rice, 70 % milled rice and brown rice.

## Discussion

### Main findings

To our knowledge, this is the first comprehensive study of monetary diet cost in relation to food and nutrient intakes in middle-aged and older Japanese women. Consistent with findings from Japanese female dietetic students and pregnant women^(^^27^^–^^29^^)^, we found that monetary diet cost was positively associated with not only healthy dietary components (such as fruits, vegetables, fish and shellfish, dietary fibre, and key vitamins and minerals), but also less healthy components (such as saturated fat and Na) across all generations of Japanese women. The major contributors to monetary diet cost were meat, total vegetables, sugar and confectioneries, fish and shellfish, white rice, and green and black tea, which are generally consistent with previous Japanese studies^(^^27^^–^^29^^,^^39^^)^. These foods were also major contributors to the inter-individual variation in monetary diet cost, which has not been investigated in previous studies.

### Comparison with previous studies

Previous studies from Western countries have consistently shown the positive association between monetary diet cost and measures of diet quality such as energy density^(^^13^^)^ and the Healthy Eating Index^(^^3^^,^^4^^,^^16^^,^^18^^)^. In a representative sample of US adults, higher monetary diet cost was associated with higher consumption of vegetables, fruits, whole grains and seafood and lower consumption of refined grains, solid fat, alcohol and added sugars^(^^16^^,^^18^^)^. A study in young and elderly people in the Netherlands also showed that those with higher energy-dense diets consumed less of fruits and vegetables and had a lower diet cost^(^^13^^)^. Relatively similar associations between monetary diet cost and dietary intakes have been observed with^(^^12^^,^^15^^–^^17^^,^^19^^,^^22^^)^ and without^(^^6^^,^^10^^,^^13^^,^^14^^,^^21^^)^ adjustment for EI, with a few exceptions^(^^46^^)^. These findings from Western countries are somewhat at odds with the present and previous Japanese studies^(^^27^^–^^29^^)^. In this study, while several ‘healthy’ foods (such as fruits, vegetables, and fish and shellfish) were major contributors to and positively associated with monetary diet cost, not only foods considered ‘unhealthy’ (such as sugar and confectionaries) but also several beverages (tea, coffee, juice and alcoholic beverages) showed similar associations with monetary diet cost. Thus, it appears that Japanese women spend more money not only on expensive but healthy foods (such as fruits, vegetables, and fish and shellfish) but also on foods consumed for pleasure or discretionary foods (such as sugar and confectionaries and beverages). This may, at least partly, explain the favourable and unfavourable characteristics of diet with higher monetary diet cost.

Only staple foods (foods with high carbohydrate contents), particularly white rice, were inversely associated with monetary diet cost. For Japanese people, white rice is a major staple food which is consumed at almost every meal, accompanied by a main and several side dishes consisting mainly of fish and shellfish, meat, eggs, vegetables and pulses. Nevertheless, white rice is inexpensive compared with the component foods of main and side dishes in Japan (which may explain the relatively small contribution of white rice to monetary diet cost). It is also probable that persons with limited money available for foods consume mainly white rice and have limited variety with respect to main and side dishes, while the reverse may be true for those with more income to spend on food. This hypothesis has some support in the observed increasing consumption of other foods such as meat, vegetables, fish and shellfish, and pulses with the increase in the Gross National Product of Japan from 1955 to 1998^(^^47^^)^. In addition, the National Health and Nutrition Survey in Japan has reported that lower household income is associated with higher intake of grains and lower intake of vegetables and meat^(^^48^^)^.

Across the generations we found that monetary diet cost was positively associated not only with intakes of nutrients such as dietary fibre and key vitamins and minerals, but also with intakes of nutrients such as saturated fat and Na and cholesterol and alcohol intakes, which is consistent with previous Japanese studies^(^^27^^,^^29^^)^. Conversely, Western studies have consistently shown that higher diet cost is associated with higher diet quality^(^^6^^,^^15^^)^. For example, a French study showed that higher monetary diet cost was associated with higher intakes of vitamins C, D and E, β-carotene, folates and Fe^(^^6^^)^. A study in low-income women in California also showed that higher monetary diet cost was related to lower intakes of saturated fat and higher intakes of vitamins A and C^(^^15^^)^. The higher fat (and thus lower carbohydrate) intake with increasing monetary diet cost observed here seemed to be due largely to decreasing consumption of white rice; in support of this, white rice intake was negatively correlated with the intake of total and saturated fats and cholesterol (Pearson correlation coefficient −0·17 to −0·70) and positively with carbohydrate intake (0·49 to 0·65). The higher intake of Na with increasing monetary diet cost observed in this study might be due to higher intakes of vegetables, meat, and fish and shellfish, because in Japan these foods are usually accompanied by seasonings with salty taste, such as salt, soya sauce, miso (fermented soyabean paste) and dressings. Actually, intakes of these foods were positively correlated with Na intake in the present study (Pearson correlation coefficient 0·08 to 0·61). The positive but unfavourable association between monetary diet cost and Na intake has also been observed in the USA^(^^16^^–^^18^^)^ and Spain^(^^10^^)^ (not reported from other countries). Thus, reducing Na intake while maintaining a healthy diet may be challenging not only in Japan but also in other affluent societies.

Despite the large difference in the association of monetary diet cost with dietary intake between Japan and Western countries, positive associations between socio-economic status and monetary diet cost have been consistently observed not only in previous Western studies^(^^7^^,^^8^^,^^21^^–^^23^^)^ but also in the present Japanese study, where education was positively associated with monetary diet cost in middle-aged and older women. This suggests that monetary diet cost is country specific. It would also be of interest whether the present findings are similarly observed in other Asian populations.

### Strengths and limitations

The major strength of the present study is a comprehensive investigation on the associations between monetary diet cost and food and nutrient intakes in three generations of women who lived over a wide geographical range of Japan and had a variety of dietary and lifestyle pattern. However, there are also several limitations. First, all our participants were female dietetic students and their mothers and grandmothers/acquaintances, and thus not a random sample of Japanese women. Given that not all Japanese adolescents enter college or university (enrollment ratio = 57 %)^(^^49^^)^, the participants in this study may therefore have a relatively high socio-economic status. Additionally, although the present survey was carried out in most institutions within 1 month after the dietetic course began to minimise the influence of dietetic education, nutritional knowledge of dietetic students and their food choices are probably different from the general population or they may be more interested in or more conscious of their diet. Furthermore, the response rate was not high enough, particularly in the older generation, which may cause self-selection bias, and thus dietary habits in the present population may be healthier than those in the general population. Thus, the present results might not be generalisable to the general Japanese population, particularly for the young generation. Additionally, because the present and previous Japanese studies^(^^27^^–^^29^^)^ included only women, it is unknown whether the associations between monetary diet cost and dietary intake observed in women are similarly observed in men.

Second, all self-report dietary assessment methods are subject to both random and systematic measurement errors. To minimise them, we assessed the dietary habits during the preceding month using well-established dietary assessment questionnaires with reasonable validity in terms of commonly studied nutritional factors (DHQ and BDHQ)^(^^31^^–^^33^^)^, as well as the use of energy-adjusted dietary variables. Additionally, as mentioned above, exclusion of EI misreporters did not change the results of almost all analyses, which may support the robustness of the present finding.

In the absence of actual food expenditure data, food price was derived from the National Retail Price Survey and websites of nationally distributed supermarket and fast-food restaurant chains. As this procedure gives only an approximation of actual diet costs, the results of the present study should be interpreted with caution, although a similar methodology has been used in many previous studies^(^^3^^,^^4^^,^^6^^,^^8^^,^^11^^,^^13^^)^. Nonetheless, the satisfactory reliability of the DHQ^(^^39^^)^ and BDHQ (S Sasaki and K Murakami, unpublished results) regarding monetary diet cost against 16-d weighed dietary record as described above may provide some assurance.

### Conclusion

Contrary to Western populations, monetary diet cost was positively associated not only with healthier dietary components (such as fruits, vegetables, fish and shellfish, dietary fibre, and key vitamins and minerals) but also with less healthy components (such as saturated fat and Na) in all three generations of Japanese women. The major contributors to monetary diet cost were meat, total vegetables, sugar and confectioneries, fish and shellfish, white rice, and green and black tea, which were also major contributors to the inter-individual variation in monetary diet cost. Because the relationship between food cost and dietary intake is an important health topic, further research in other populations, particularly men and children, is warranted.

## Supplementary material

For supplementary material accompanying this paper visit http://dx.doi.org/10.1017/jns.2017.18. click here to view supplementary material
