# Supplementary material for: Nutritional correlates of monetary diet cost in young, middle-aged and older Japanese women
Source: J Nutr Sci. 2017 May 22;6:e22. doi: 10.1017/jns.2017.18 (PMC5468738; doi:10.1017/jns.2017.18)
Supplement: Supplementary file 1 [file S2048679017000180sup001.doc]

**Supplementary Table S1.** Monetary cost and percentage contribution of each food item to monetary diet cost in the comprehensive diet history questionnaire in young and middle-aged Japanese women*

(Mean values and standard deviations)

Monetary cost Young (*n* 3963) Middle-aged (*n* 3800)

|  | (Japanese yen/100 g) | Mean | SD | Mean | SD |
| --- | --- | --- | --- | --- | --- |
| White rice White rice | 21 | 7.28 | 4.90 | 5.79 | 3.93 |
| Other grains  White rice mixed with barley | 16 | 0.12 | 0.88 | 0.07 | 0.59 |
| White rice with germ | 21 | 0.16 | 1.08 | 0.13 | 0.90 |
| Half-milled rice | 21 | 0.13 | 1.17 | 0.08 | 0.82 |
| 70 %-milled rice | 21 | 0.12 | 1.07 | 0.20 | 1.23 |
| Brown rice | 21 | 0.20 | 1.20 | 0.15 | 0.93 |
| Noodles  Japanese noodles (buckwheat | 26 | 0.69 | 0.92 | 0.64 | 0.76 |
| and Japanese wheat noodles) Instant noodles | 54 | 0.80 | 1.50 | 0.69 | 1.16 |
| Chinese noodles | 54 | 0.48 | 0.98 | 0.48 | 0.83 |
| Spaghetti | 20 | 0.55 | 0.76 | 0.36 | 0.42 |
| Bread  White bread | 42 | 1.18 | 1.39 | 1.09 | 1.19 |
| Butter roll | 42 | 0.28 | 0.61 | 0.27 | 0.51 |
| Croissant | 42 | 0.29 | 0.57 | 0.26 | 0.48 |
| Other grain products Pizza | 42 | 0.20 | 0.44 | 0.18 | 0.39 |
| Japanese-style pancakes | 97 | 0.73 | 1.20 | 0.67 | 1.13 |
| Cornflakes | 99 | 0.23 | 0.73 | 0.11 | 0.52 |
| Pulses Peanuts | 107 | 0.08 | 0.24 | 0.14 | 0.32 |
| Other nuts | 107 | 0.07 | 0.25 | 0.09 | 0.29 |
| Tofu (i.e., soybean curd) | 27 | 0.56 | 0.58 | 0.70 | 0.56 |
| Tofu products | 148 | 0.37 | 0.78 | 0.49 | 0.92 |
| Natto (i.e., fermented soybeans) | 89 | 0.66 | 1.06 | 0.81 | 1.06 |
| Boiled beans | 119 | 0.43 | 0.76 | 0.49 | 0.78 |
| Miso as seasoning | 34 | 0.10 | 0.28 | 0.15 | 0.34 |
| Miso for miso soup | 34 | 0.23 | 0.23 | 0.22 | 0.19 |
| Soy milk | 20 | 0.26 | 0.93 | 0.23 | 0.87 |
| Potatoes French fries | 181 | 0.69 | 0.87 | 0.36 | 0.51 |
| Potatoes | 30 | 0.52 | 0.57 | 0.52 | 0.46 |
| Sweet potatoes, yams, and taro | 61 | 0.48 | 0.58 | 0.51 | 0.65 |
| Konnyaku (i.e., devil's tongue jelly) | 43 | 0.12 | 0.16 | 0.14 | 0.15 |
| Sugar and confectioneries Jam and marmalade | 123 | 0.30 | 0.70 | 0.28 | 0.59 |
| Sugar for coffee and black tea | 18 | 0.04 | 0.10 | 0.06 | 0.13 |
| Sugar used during cooking | 18 | 0.15 | 0.08 | 0.16 | 0.06 |
| Japanese bread with a sweet filling | 82 | 1.49 | 1.92 | 1.37 | 1.78 |
| Pancakes | 38 | 0.17 | 0.44 | 0.09 | 0.26 |
| Potato chips | 149 | 0.24 | 0.40 | 0.17 | 0.31 |
| Rice crackers | 129 | 0.36 | 0.63 | 0.80 | 1.10 |
| Snacks made from wheat flour | 149 | 0.66 | 1.06 | 0.43 | 0.70 |
| Japanese sweets with azuki beans | 168 | 0.45 | 0.58 | 0.73 | 0.87 |
| Japanese sweets | 167 | 0.20 | 0.42 | 0.28 | 0.47 |
| without azuki beans Cakes | 214 | 1.71 | 1.94 | 1.46 | 1.53 |
| Cookies and biscuits | 131 | 0.72 | 0.92 | 0.59 | 0.82 |
| Chocolates | 135 | 1.12 | 1.56 | 0.86 | 1.14 |
| Candies, caramels, and chewing gum | 329 | 1.99 | 2.83 | 1.28 | 2.21 |
| Jellies | 59 | 0.49 | 0.84 | 0.26 | 0.54 |
| Doughnuts | 157 | 0.71 | 1.26 | 0.58 | 1.08 |
| Ice cream (regular) | 90 | 1.34 | 2.96 | 0.56 | 1.63 |
| Ice cream (premium) | 209 | 0.27 | 2.02 | 0.21 | 1.56 |
| Ice cream (unspecified varieties) | 150 | 1.78 | 3.20 | 1.11 | 2.09 |

**Supplementary Table S1.** Continued

Monetary cost Young (*n* 3963) Middle-aged (*n* 3800)

|  | (Japanese yen/100 g) | Mean | SD | Mean | SD |
| --- | --- | --- | --- | --- | --- |
| Fat and oil Butter | 157 | 0.14 | 0.26 | 0.11 | 0.21 |
| Margarine | 55 | 0.07 | 0.14 | 0.11 | 0.17 |
| Mayonnaise | 56 | 0.32 | 0.50 | 0.31 | 0.47 |
| Salad dressing | 107 | 0.77 | 1.14 | 0.53 | 0.85 |
| Oil used during cooking | 29 | 0.37 | 0.14 | 0.33 | 0.11 |
| Fruit Raisins | 110 | 0.05 | 0.22 | 0.06 | 0.31 |
| Canned fruits | 34 | 0.05 | 0.14 | 0.03 | 0.09 |
| Oranges | 52 | 0.52 | 1.04 | 0.72 | 1.26 |
| Bananas | 34 | 0.43 | 0.88 | 0.69 | 1.02 |
| Apples | 55 | 0.60 | 1.24 | 0.54 | 1.13 |
| Strawberries | 150 | 1.47 | 2.15 | 1.45 | 1.99 |
| Grapes | 140 | 0.10 | 0.46 | 0.06 | 0.34 |
| Peaches | 100 | 0.06 | 0.25 | 0.01 | 0.11 |
| Pears | 58 | 0.02 | 0.43 | 0.00 | 0.05 |
| Persimmons | 62 | 0.01 | 0.09 | 0.00 | 0.05 |
| Kiwi fruits | 85 | 0.18 | 0.72 | 0.17 | 0.53 |
| Melons | 90 | 0.05 | 0.22 | 0.05 | 0.24 |
| Watermelons | 45 | 0.01 | 0.13 | 0.02 | 0.10 |
| Total vegetable Carrots | 37 | 0.52 | 0.48 | 0.55 | 0.44 |
| Pumpkins | 36 | 0.24 | 0.35 | 0.23 | 0.30 |
| Tomatoes | 63 | 1.16 | 1.41 | 0.95 | 1.10 |
| Green peppers | 83 | 0.45 | 0.57 | 0.42 | 0.44 |
| Broccoli | 81 | 0.75 | 1.07 | 0.77 | 0.92 |
| Green leafy vegetables | 122 | 3.52 | 3.72 | 3.78 | 3.51 |
| Salted pickled plums | 237 | 0.31 | 0.67 | 0.33 | 0.64 |
| Cabbage | 23 | 0.69 | 0.69 | 0.62 | 0.51 |
| Cucumbers | 53 | 0.53 | 0.63 | 0.55 | 0.52 |
| Lettuce | 55 | 0.56 | 0.67 | 0.52 | 0.51 |
| Chinese cabbage | 21 | 0.19 | 0.29 | 0.18 | 0.27 |
| Bean sprouts | 22 | 0.23 | 0.29 | 0.23 | 0.21 |
| Radishes | 22 | 0.20 | 0.25 | 0.23 | 0.24 |
| Onions | 25 | 0.49 | 0.43 | 0.55 | 0.41 |
| Cauliflower | 90 | 0.05 | 0.29 | 0.05 | 0.25 |
| Eggplants | 64 | 0.29 | 0.50 | 0.29 | 0.41 |
| Burdock | 77 | 0.28 | 0.38 | 0.32 | 0.39 |
| Lotus root | 146 | 0.22 | 0.39 | 0.24 | 0.35 |
| Salted pickles (leafy vegetables) | 54 | 0.16 | 0.36 | 0.19 | 0.43 |
| Salted pickles (others) | 86 | 0.34 | 0.72 | 0.63 | 1.04 |
| Mushrooms | 158 | 1.77 | 1.87 | 2.29 | 2.01 |
| Wakame and hijiki seaweed | 119 | 1.53 | 1.84 | 1.55 | 1.53 |
| Laver (i.e., dried, edible seaweed) | 1787 | 0.32 | 0.48 | 0.40 | 0.46 |
| Fruit and vegetable juice Vegetable juice | 25 | 0.67 | 2.04 | 0.35 | 1.14 |
| Fruit juice (100 %) | 22 | 0.60 | 1.51 | 0.23 | 0.67 |
| Tomato juice | 33 | 0.05 | 0.44 | 0.12 | 0.83 |
| Alcoholic beverages Beer | 59 | 0.06 | 1.15 | 3.77 | 8.78 |
| Sake | 81 | 0.00 | 0.04 | 0.15 | 1.39 |
| Shochu | 87 | 0.01 | 0.42 | 0.32 | 1.91 |
| Shochu mixed with water | 37 | 0.04 | 0.35 | 0.18 | 0.89 |
| or a carbonated beverage Whiskey | 139 | 0.00 | 0.07 | 0.02 | 0.29 |
| Wine | 116 | 0.01 | 0.16 | 0.38 | 1.99 |
| Green and black tea  Green, barley, and oolong tea | 12 | 7.54 | 6.39 | 5.96 | 4.94 |
| (including other Chinese tea)  Black tea | 3 | 0.18 | 0.49 | 0.16 | 0.38 |

**Supplementary Table S1.** Continued

Monetary cost Young (*n* 3963) Middle-aged (*n* 3800)

|  | (Japanese yen/100 g) | Mean | SD | Mean | SD |
| --- | --- | --- | --- | --- | --- |
| Coffee Coffee | 13 | 0.50 | 1.53 | 4.10 | 3.75 |
| Soft drinks  Fruit juice excluding 100 % juice | 22 | 0.33 | 1.09 | 0.10 | 0.48 |
| Cocoa | 11 | 0.15 | 0.58 | 0.05 | 0.27 |
| Lactic acid bacteria beverages | 15 | 0.19 | 0.49 | 0.14 | 0.39 |
| Sugar-sweetened soft drinks | 24 | 1.21 | 2.89 | 0.51 | 1.57 |
| Sugar-free soft drinks | 24 | 0.33 | 1.61 | 0.21 | 1.28 |
| Nutritional supplement drinks | 92 | 0.25 | 1.28 | 0.56 | 1.91 |
| Fish and shellfish Dried fish | 155 | 0.69 | 1.11 | 1.08 | 1.37 |
| Small fish with bones | 285 | 0.33 | 0.62 | 0.50 | 0.80 |
| Canned tuna | 192 | 0.48 | 0.78 | 0.44 | 0.67 |
| Eel | 619 | 0.26 | 0.86 | 0.30 | 0.82 |
| White meat fish | 258 | 1.97 | 2.06 | 1.73 | 1.85 |
| Oily fish | 159 | 1.28 | 1.38 | 1.60 | 1.51 |
| Red meat fish | 265 | 2.34 | 2.29 | 2.43 | 2.08 |
| Ground fish meat products | 121 | 0.57 | 0.79 | 0.72 | 0.77 |
| Shrimp and crab | 295 | 1.48 | 1.69 | 1.37 | 1.41 |
| Squid and octopus | 204 | 0.90 | 1.05 | 1.00 | 0.95 |
| Oysters | 469 | 0.11 | 0.43 | 0.14 | 0.42 |
| Other shellfish | 216 | 0.40 | 0.70 | 0.34 | 0.42 |
| Fish eggs | 575 | 0.58 | 1.09 | 0.57 | 0.89 |
| Boiled fish and shellfish | 280 | 0.26 | 0.74 | 0.40 | 0.80 |
| in soy sauce Salted fish intestines | 188 | 0.03 | 0.17 | 0.03 | 0.16 |
| Meat  Ground beef and pork | 283 | 2.97 | 2.98 | 2.21 | 2.21 |
| Chicken | 150 | 3.04 | 2.52 | 2.70 | 2.08 |
| Pork | 196 | 4.35 | 3.37 | 4.86 | 3.39 |
| Beef | 375 | 4.37 | 3.93 | 3.15 | 3.21 |
| Liver | 120 | 0.10 | 0.30 | 0.09 | 0.25 |
| Ham and sausages | 212 | 1.71 | 1.83 | 1.42 | 1.39 |
| Bacon | 212 | 0.66 | 0.97 | 0.56 | 0.70 |
| Eggs  Eggs | 37 | 1.67 | 1.55 | 1.31 | 0.97 |
| Dairy products Sweetened yogurt | 39 | 0.77 | 1.59 | 0.59 | 1.36 |
| Non-sweetened yogurt | 39 | 0.23 | 1.06 | 0.31 | 1.14 |
| Moderately sweetened yogurt | 39 | 0.53 | 1.51 | 0.64 | 1.62 |
| Cheese | 148 | 0.62 | 1.02 | 0.60 | 1.03 |
| Cottage cheese | 148 | 0.06 | 0.27 | 0.03 | 0.19 |
| Low-fat milk | 20 | 0.51 | 1.65 | 0.45 | 1.38 |
| Full-fat milk | 20 | 1.17 | 2.30 | 1.35 | 2.05 |
| Others Ketchup | 52 | 0.09 | 0.14 | 0.05 | 0.10 |
| Fat-free salad dressing | 107 | 0.39 | 0.93 | 0.34 | 0.70 |
| Table salt | 11 | 0.01 | 0.02 | 0.01 | 0.01 |
| Salt used during cooking | 11 | 0.04 | 0.02 | 0.04 | 0.01 |
| Soy sauce | 28 | 0.18 | 0.09 | 0.17 | 0.08 |
| Curry and roux in stew | 93 | 0.02 | 0.02 | 0.01 | 0.01 |
| Cream or creamer added to coffee | 207 | 0.27 | 0.79 | 0.72 | 1.33 |
| Corn soup | 7 | 0.02 | 0.10 | 0.01 | 0.10 |
| Chinese soup | 7 | 0.02 | 0.06 | 0.01 | 0.06 |
| Soup consumed with noodles | 0.1 | 0.01 | 0.01 | 0.01 | 0.01 |
| Water for miso soup | 0 | 0.00 | 0.00 | 0.00 | 0.00 |
| Nutritional supplement bars | 259 | 0.14 | 0.84 | 0.02 | 0.27 |
| Artificial sweeteners | 473 | 0.01 | 0.08 | 0.03 | 0.17 |
| Drinking water | 0 | 0.00 | 0.00 | 0.00 | 0.00 |

1 Japanese yen = 0.0099 US dollars = 0.0088 Euros = 0.0074 British pounds (September 2016).

**Supplementary Table S2.** Monetary cost and percentage contribution of each food item to monetary diet cost in the brief-type diet history questionnaire in older Japanese women*

(Mean values and standard deviations)

Monetary cost Older (*n* 2211)

|  | (Japanese yen/100 g) | Mean | SD |
| --- | --- | --- | --- |
| White rice White rice | 21 | 5.97 | 3.36 |
| Noodle  Buckwheat noodles | 25 | 0.25 | 0.37 |
| Japanese wheat noodles | 29 | 0.64 | 0.69 |
| Instant noodles and | 54 | 0.48 | 0.81 |
| Chinese noodles  Spaghetti and macaroni | 20 | 0.18 | 0.24 |
| Bread  Breads (including white bread and | 62 | 2.20 | 1.87 |
| Japanese bread with a sweet filling)  Pulses  Tofu (i.e., soybean curd) and tofu products | 30 | 1.59 | 1.05 |
| Natto (i.e., fermented soybeans) | 89 | 1.19 | 1.35 |
| Miso for miso soup | 34 | 0.27 | 0.19 |
| Potatoes  Potatoes (all varieties) | 42 | 2.19 | 1.60 |
| Sugar and confectioneries Cakes, cookies, and biscuits | 207 | 5.60 | 5.48 |
| Japanese sweets | 143 | 1.74 | 1.89 |
| Rice crackers, rice cakes, and | 129 | 1.97 | 2.05 |
| Japanese-style pancakes Ice cream | 150 | 1.13 | 2.40 |
| Sugar for coffee and black tea | 18 | 0.10 | 0.08 |
| Sugar used during cooking | 18 | 0.06 | 0.03 |
| Fat and oil  Mayonnaise and salad dressing | 73 | 0.40 | 0.32 |
| Oil used during cooking | 29 | 0.24 | 0.12 |
| Fruit  Citrus fruit | 52 | 2.14 | 1.95 |
| Strawberries, persimmons, and kiwi fruits | 98 | 1.87 | 2.21 |
| Other fruit | 69 | 2.65 | 2.37 |
| Total vegetable  Salted green and yellow vegetable pickles | 54 | 0.67 | 0.60 |
| Other salted vegetables | 86 | 1.16 | 1.11 |
| (excluding salted pickled plums)  Raw vegetables used in salad | 43 | 1.15 | 0.83 |
| (e.g., cabbage and lettuce)  Green leafy vegetables including broccoli | 97 | 5.03 | 3.41 |
| Cabbage and Chinese cabbage | 21 | 0.92 | 0.60 |
| Carrots and pumpkins | 32 | 0.74 | 0.51 |
| Radishes and turnips | 20 | 0.54 | 0.45 |
| Other root vegetables | 35 | 1.33 | 0.85 |
| (e.g., onions, burdock, and lotus root) Tomatoes, tomato ketchup, boiled tomato, | 63 | 1.25 | 1.40 |
| and stewed tomato Mushrooms (all varieties) | 158 | 1.95 | 1.42 |
| Seaweeds (all varieties) | 119 | 1.83 | 1.36 |
| Fruit and vegetable juice  Fruit and vegetable juice (100%) | 24 | 0.79 | 1.62 |
| Alcoholic beverages Sake | 81 | 0.18 | 1.34 |
| Beer | 59 | 0.56 | 2.98 |
| Shochu and Shochu mixed with water | 87 | 0.14 | 0.96 |
| or a carbonated beverage  Whiskey | 139 | 0.01 | 0.22 |
| Wine | 116 | 0.10 | 0.92 |
| Green and black tea Green tea | 13 | 4.80 | 3.45 |
| Black and oolong tea (including other Chinese | 3 | 0.17 | 0.35 |
| Coffee Coffee | 13 | 1.63 | 1.92 |
| Soft drinks  Cola and sugar-sweetened soft drinks | 24 | 0.50 | 1.61 |

**Supplementary Table S2.** Continued

Monetary cost Elderly (*n* 2211)

|  | (Japanese yen/100 g) | Mean | SD |
| --- | --- | --- | --- |
| Fish and shellfish  Squid, octopus, shrimp, and clam | 226 | 3.19 | 2.87 |
| Small fish with bones | 285 | 4.88 | 5.09 |
| Canned tuna | 192 | 0.56 | 0.89 |
| Dried fish and salted fish (including salted | 246 | 4.74 | 3.78 |
| salted salmon, and dried horse mackerel) Oily fish (including sardines, mackerel, saury, | 226 | 4.20 | 3.21 |
| amberjack, herring, eel, and fatty tuna) Non-oily fish (including salmon, trout, | 258 | 4.84 | 3.63 |
| white meat fish, freshwater fish, and bonito)  Meat  Chicken (including ground chicken) | 120 | 2.35 | 1.89 |
| Pork and beef (including ground and beef) | 283 | 6.98 | 4.73 |
| Ham, sausages, and bacon | 212 | 1.29 | 1.45 |
| Liver | 120 | 0.12 | 0.30 |
| Eggs  Eggs | 37 | 1.21 | 0.79 |
| Dairy products  Low-fat milk and yogurt | 20 | 0.81 | 1.39 |
| Full-fat milk and yogurt | 20 | 1.46 | 1.58 |
| Others  Salt used during cooking | 0.2 | 0.03 | 0.01 |
| Soy sauce | 11 | 0.02 | 0.01 |
| Soup consumed with noodles | 11 | 0.01 | 0.01 |

1 Japanese yen = 0.0099 US dollars = 0.0088 Euros = 0.0074 British pounds (September 2016).

**Supplementary Table S3.** Monetary diet cost according to categories of selected characteristics in young, middle-aged, and older Japanese women*

(Numbers and percentages; mean values and standard deviations)

|  |  |  | Young (*n* 3963) |  |  | Middle-aged (*n* 3800) |  |  | Older (*n* 2211) |
| --- | --- | --- | --- | --- | --- | --- | --- | --- | --- |

Monetary diet cost Monetary diet cost Monetary diet cost

(Japanese yen/4184 kJ) (Japanese yen/4184 kJ) (Japanese yen/4184 kJ)

|  | *n* | % | Mean | SD | *P* | *n* | % | Mean | SD | *P* | *n* | % | Mean | SD | *P* |
| --- | --- | --- | --- | --- | --- | --- | --- | --- | --- | --- | --- | --- | --- | --- | --- |
| Survey year |  |  |  |  | 0.19 |  |  |  |  | 0.66 |  |  |  |  | <0.0001 |
| 2011 | 2461 | 62.1 | 481 | 99 |  | 2311 | 60.8 | 530 | 101 |  | 1408 | 63.7 | 618 | 104 |  |
| 2012 | 1502 | 37.9 | 477 | 95 |  | 1489 | 39.2 | 532 | 101 |  | 803 | 36.3 | 648 | 105 |  |
| Residential block |  |  |  |  | <0.0001 |  |  |  |  | 0.37 |  |  |  |  | <0.0001 |
| Hokkaido and Tohoku | 379 | 9.6 | 454 | 95 |  | 383 | 10.1 | 539 | 108 |  | 209 | 9.5 | 624 | 95 |  |
| Kanto | 1144 | 28.9 | 483 | 93 |  | 1074 | 28.3 | 528 | 95 |  | 547 | 24.7 | 659 | 107 |  |
| Hokuriku and Tokai | 808 | 20.4 | 485 | 100 |  | 840 | 22.1 | 532 | 104 |  | 539 | 24.4 | 615 | 104 |  |
| Kinki | 480 | 12.1 | 486 | 97 |  | 494 | 13.0 | 525 | 103 |  | 273 | 12.4 | 621 | 110 |  |
| Chugoku and Shikoku | 557 | 14.1 | 476 | 103 |  | 520 | 13.7 | 532 | 99 |  | 359 | 16.2 | 623 | 107 |  |
| Kyushu | 595 | 15.0 | 478 | 97 |  | 489 | 12.9 | 534 | 99 |  | 284 | 12.8 | 618 | 94 |  |
| Living status |  |  |  |  |  |  |  |  |  |  |  |  |  |  | 0.999 |
| Living alone | 923 | 23.3 | 431 | 94 | <0.0001 | --- | --- | --- | --- |  | 355 | 16.1 | 629 | 115 |  |
| Living with family | 2910 | 73.4 | 495 | 94 |  | --- | --- | --- | --- |  | 1856* | 83.9 | 629 | 103 |  |
| Living with others | 130 | 3.3 | 461 | 92 |  | --- | --- | --- | --- |  | --- | --- |  |  |  |
| Size of residential area |  |  |  |  | 0.19 |  |  |  |  | 0.004 |  |  |  |  | 0.006 |
| Town and village | 310 | 7.8 | 479 | 97 |  | 384 | 10.1 | 519 | 99 |  | 244 | 11.0 | 609 | 101 |  |
| City with a population <1million | 2914 | 73.5 | 478 | 98 |  | 2821 | 74.2 | 531 | 101 |  | 1682 | 76.1 | 631 | 107 |  |
| City with a population ≥1 million | 739 | 18.7 | 485 | 95 |  | 595 | 15.7 | 540 | 102 |  | 285 | 12.9 | 635 | 102 |  |
| Weight status† |  |  |  |  | 0.14 |  |  |  |  | 0.55 |  |  |  |  | 0.004 |
| Underweight | 685 | 17.3 | 486 | 104 |  | 320 | 8.4 | 537 | 109 |  | 167 | 7.6 | 629 | 108 |  |
| Normal weight | 3015 | 76.1 | 477 | 96 |  | 2904 | 76.4 | 531 | 101 |  | 1555 | 70.3 | 634 | 105 |  |
| Overweight | 263 | 6.6 | 481 | 97 |  | 576 | 15.2 | 529 | 96 |  | 489 | 22.1 | 615 | 104 |  |
| Current smoking |  |  |  |  | 0.60 |  |  |  |  | <0.0001 |  |  |  |  | 0.68 |
| Yes | 7 | 0.2 | 498 | 175 |  | 290 | 7.6 | 560 | 124 |  | 59 | 2.7 | 635 | 116 |  |
| No | 3956 | 99.8 | 479 | 97 |  | 3510 | 92.4 | 529 | 98 |  | 2152 | 97.3 | 629 | 105 |  |
| Current alcohol drinking |  |  |  |  | 0.08 |  |  |  |  | <0.0001 |  |  |  |  | <0.0001 |
| Yes | 237 | 6.0 | 490 | 100 |  | 1928 | 50.7 | 506 | 90 |  | 440 | 19.9 | 652 | 108 |  |
| No | 3726 | 94.0 | 478 | 97 |  | 1872 | 49.3 | 557 | 105 |  | 1771 | 80.1 | 623 | 104 |  |
| Dietary supplement use |  |  |  |  | <0.0001 |  |  |  |  | <0.0001 |  |  |  |  | 0.0006 |
| Yes | 257 | 6.5 | 512 | 103 |  | 982 | 74.2 | 526 | 100 |  | 664 | 30.0 | 641 | 106 |  |
| No | 3706 | 93.5 | 477 | 97 |  | 2818 | 25.8 | 544 | 101 |  | 1547 | 70.0 | 624 | 105 |  |
| Medication use |  |  |  |  | <0.0001 |  |  |  |  | 0.11 |  |  |  |  | 0.041 |
| Yes | 427 | 10.8 | 499 | 103 |  | 962 | 74.7 | 529 | 99 |  | 1700 | 76.9 | 627 | 104 |  |
| No | 3536 | 89.2 | 477 | 97 |  | 2838 | 25.3 | 535 | 105 |  | 511 | 23.1 | 637 | 111 |  |

**Supplementary Table S3.** Continued

|  |  |  | Young (*n* 3963) |  |  | Middle-aged (*n* 3800) |  |  | Older (*n* 2211) |
| --- | --- | --- | --- | --- | --- | --- | --- | --- | --- |

Monetary diet cost Monetary diet cost Monetary diet cost (Japanese yen/4184 kJ) (Japanese yen/4184 kJ) (Japanese yen/4184 kJ)

|  | *n* | % | Mean | SD | *P* | *n* | % | Mean | SD | *P* | *n* | % | Mean | SD | *P* |
| --- | --- | --- | --- | --- | --- | --- | --- | --- | --- | --- | --- | --- | --- | --- | --- |
| Eating out |  |  |  |  | 0.02 |  |  |  |  | 0.64 |  |  |  |  |  |
| ≤3 times/month | 1685 | 42.5 | 474 | 102 |  | 2368 | 62.3 | 531 | 103 |  | --- | --- | --- | --- |  |
| Once per week | 826 | 20.8 | 484 | 96 |  | 656 | 17.3 | 528 | 94 |  | --- | --- | --- | --- |  |
| 2-3 times/week | 790 | 19.9 | 485 | 92 |  | 445 | 11.7 | 536 | 95 |  | --- | --- | --- | --- |  |
| ≥4 times/week | 662 | 16.7 | 479 | 95 |  | 331 | 8.7 | 529 | 101 |  | --- | --- | --- | --- |  |
| Occupation |  |  |  |  |  |  |  |  |  | 0.047 |  |  |  |  |  |
| Housewife | --- | --- | --- | --- |  | 747 | 19.7 | 536 | 94 |  | --- | --- | --- | --- |  |
| Part-time job | --- | --- | --- | --- |  | 1687 | 44.4 | 527 | 102 |  | --- | --- | --- | --- |  |
| Full-time job | --- | --- | --- | --- |  | 1366 | 36.0 | 533 | 102 |  | --- | --- | --- | --- |  |
| Physical activity |  |  |  |  | <0.0001 |  |  |  |  | 0.46 |  |  |  |  | <0.0001 |
| Quartile 1 (lowest) | 978 | 24.7 | 466 | 91 |  | 946 | 24.9 | 527 | 106 |  | 547 | 24.7 | 605 | 108 |  |
| Quartile 2 | 1017 | 25.7 | 471 | 96 |  | 950 | 25.0 | 533 | 95 |  | 558 | 25.2 | 628 | 100 |  |
| Quartile 3 | 1002 | 25.3 | 485 | 101 |  | 953 | 25.1 | 533 | 101 |  | 553 | 25.0 | 642 | 109 |  |
| Quartile 4 | 966 | 24.4 | 495 | 99 |  | 951 | 25.0 | 531 | 101 |  | 553 | 25.0 | 642 | 102 |  |
| Education |  |  |  |  |  |  |  |  |  | 0.0002 |  |  |  |  | <0.0001 |
| Low‡ | --- | --- | --- | --- |  | 1823 | 48.0 | 525 | 104 |  | 990 | 44.8 | 618 | 107 |  |
| Middle§ | --- | --- | --- | --- |  | 1425 | 35.5 | 533 | 97 |  | 1005 | 45.5 | 634 | 102 |  |
| High|| | --- | --- | --- | --- |  | 552 | 14.5 | 544 | 99 |  | 216 | 9.8 | 655 | 106 |  |
| Current marital status |  |  |  |  |  |  |  |  |  | 0.87 |  |  |  |  | <0.0001 |
| Yes | --- | --- | --- | --- |  | 3479 | 91.6 | 532 | 120 |  | 1346 | 60.9 | 636 | 100 |  |
| No | --- | --- | --- | --- |  | 321 | 8.5 | 531 | 99 |  | 865 | 39.1 | 618 | 112 |  |
| Dietary reporting status¶ |  |  |  |  |  |  |  |  |  | 0.67 |  |  |  |  | <0.0001 |
| Under-reporting | 742 | 18.7 | 456 | 110 | <0.0001 | 291 | 7.7 | 527 | 129 |  | 141 | 6.4 | 634 | 139 |  |
| Plausible reporting | 2962 | 74.7 | 483 | 93 |  | 3085 | 81.2 | 531 | 96 |  | 1549 | 70.1 | 623 | 105 |  |
| Over-reporting | 259 | 6.5 | 501 | 103 |  | 424 | 11.2 | 534 | 112 |  | 521 | 23.6 | 646 | 95 |  |

*Including women living with others because of the small number of subjects (*n* 4). 1 Japanese yen = 0.0099 US dollars = 0.0088 Euros = 0.0074 British pounds (September 2016).

†Underweight were defined as subjects with BMI <18.5 kg/m^2^; nomal weight defined as subjects with BMI ≥18.5 to <25 kg/m^2^; overweight defined as subjects with BMI ≥ 25.0 kg/m^2^. ‡≤12 years for middle-aged women and ≤9 years for older women.

§13-15 years for middle-aged women and 10-12 years for older women.

||≥16 years for middle-aged women and ≥13 years for older women.

¶Under-reporting were defined as subjects with a ratio of reported energy intake to BMR (EI:BMR) <1.09; plausible reporting defined as subjects with EI:BMR 1.09-2.21; over-reporting defined as subjects with EI:BMR > 2.21.
